# Gut Loading of Yellow Mealworm (*Tenebrio molitor*) Larvae with Dried Carrot: Effects on Retinol, Carotenoids, Tocopherols, Fatty Acid Profile, and Oxidative Status Biomarkers

**DOI:** 10.3390/insects17101090

**Published:** 2026-10-21

**Authors:** Janusz Strychalski, Oskar Parchucik, Andrzej Gugołek

**Affiliations:** 1 Department of Fur-Bearing Animal Breeding and Game Management, University of Warmia and Mazury in Olsztyn, Oczapowskiego 2, 10-719 Olsztyn, Poland; gugolek@uwm.edu.pl; 2 Student Scientific Circle of Amateur Animal Breeders, University of Warmia and Mazury in Olsztyn, Oczapowskiego 2, 10-719 Olsztyn, Poland; oskar.parchucik@student.uwm.edu.pl

**Keywords:** feeder insects, dietary enrichment, insect nutrition, provitamin A, vitamin E, lipid peroxidation, nutrient composition

## Abstract

Gut loading may improve the nutritional value of feeder insects used for insectivorous animals. This study evaluated the effects of dried carrot inclusion in wheat-bran-based diets on the chemical composition, lipid-soluble bioactive compounds, fatty acid profile, and oxidative status of yellow mealworm (*Tenebrio molitor*) larvae. Larvae were assigned to three dietary treatments (10 replicates per treatment) and fed for 48 h with wheat bran alone (WB), 50% dried carrot and 50% wheat bran (DC50), or 75% dried carrot and 25% wheat bran (DC75). Dried carrot inclusion did not affect the proximate composition of the larvae. Retinol concentration was higher in DC75, whereas β-carotene, lutein, and zeaxanthin increased with increasing dried carrot inclusion. In contrast, α-, β-, and total tocopherol concentrations were lower in carrot-fed larvae. Dried carrot also modified the fatty acid profile, affecting several individual fatty acids as well as total polyunsaturated fatty acids (PUFA) and the n-6/n-3 PUFA ratio. Peroxidase and superoxide dismutase activities were unaffected, whereas malondialdehyde concentration was lower in DC75 than in WB. These results indicate that short-term feeding with dried carrot increases carotenoid concentrations in whole mealworm larvae and modifies selected indicators of lipid quality and oxidative status.

## Simple Summary

Yellow mealworm larvae are widely used as live feed for reptiles, amphibians, and other insect-eating animals, but their nutritional value can be improved before feeding. One practical method is gut loading, in which insects are given nutrient-rich feed shortly before they are offered to animals. This study examined whether dried carrot could be used to modify yellow mealworm larvae with nutritionally valuable compounds. Larvae were fed for 48 h with diets containing wheat bran alone or wheat bran mixed with 50% or 75% dried carrot. Adding dried carrot did not markedly change the basic nutrient composition of the larvae, but it increased their contents of beta-carotene, lutein, zeaxanthin, and retinol. It also modified the fatty acid profile, affecting the proportions of several saturated, monounsaturated, and polyunsaturated fatty acids and lowering the omega-6 to omega-3 fatty acid ratio. At the highest carrot level, a marker of lipid oxidation was also lower, although antioxidant enzyme activity was unchanged. These results indicate that short-term feeding with dried carrot can modify the nutritional composition and oxidative status of whole mealworm larvae and may help improve the nutritional value of insects used as feed for insectivorous animals.

## 1. Introduction

Yellow mealworm (*Tenebrio molitor*) is one of the most extensively studied insect species [1,2,3], is widely produced on a commercial scale [4,5], and is commonly used as a feeder insect for exotic animals, particularly reptiles and amphibians [6,7]. The nutritional quality of feeder insects may directly affect the nutrient supply and nutritional welfare of the target animal [8,9,10]. However, commercially reared feeder insects do not always provide nutrients in proportions appropriate for strict insectivores, with imbalances or relatively low concentrations reported particularly for calcium and several vitamins, including vitamins A, D, and E [10,11,12]. Dietary manipulation has been proposed as a means of improving the nutritional value of feeder insects, including through the enrichment of selected nutrients and bioactive compounds [4,13,14]. Experimental studies have further demonstrated that the nutritional composition of insects can be influenced by the composition of the diet provided during rearing [15,16,17], creating opportunities to modify the concentrations and profiles of selected nutrients. In the context of live feeder insects, a practical short-term strategy is gut loading, in which insects are offered nutrient-rich diets shortly before being consumed, allowing selected nutrients to be delivered indirectly to the target animal [9,18,19].

Among the most important lipid-soluble bioactive compounds involved in animal nutrition are carotenoids and tocopherols. Carotenoids, particularly β-carotene, function both as antioxidants and as provitamin A compounds that can be converted into retinoids (one of them is retinol) in animal organisms, thereby contributing to physiological processes such as immune function, growth, reproduction, and visual function [20]. In addition to their role as precursors of vitamin A, carotenoids act as important antioxidants protecting cells against oxidative stress and supporting immune responses in animals [21]. One of the richest natural dietary sources of carotenoids, particularly β-carotene, is carrot (*Daucus carota* L.) [22,23,24]. In addition to carotenoids, carrots contain other biologically active compounds such as vitamin C, phenolic compounds, and dietary fiber, contributing to their antioxidant potential [22,25].

Despite growing interest in modifying the nutritional value of mealworm larvae through dietary manipulation, relatively limited information is available on the extent to which dietary composition can be used to enrich larvae with specific lipid-soluble bioactive compounds [13,26]. Previous studies have demonstrated that the composition of insect diets can influence specific components of larval chemical composition [15,16,17], although such effects are not consistently observed across dietary treatments [27]. Gut loading can also be used to increase the concentrations of selected nutrients in feeder insects [11,18,19]. However, substantial amounts of carotenoids, including zeaxanthin, lycopene, and β-carotene, may be excreted in frass [26,28]. Although carrot and carrot-derived ingredients have been investigated as dietary components for *Tenebrio molitor*, previous studies have focused mainly on growth, proximate composition, antioxidant activity, or total carotenoid content. Information on the effects of dried carrot on the concentrations of retinol, β-carotene, and individual tocopherol homologues in mealworm larvae remains limited [26,27,29]. In addition, dietary manipulation may affect the fatty acid composition and oxidative status of insects, particularly when plant-derived ingredients rich in bioactive compounds are used [26,30,31]. Therefore, the aim of the present study was to evaluate the effects of diets differing in the proportion of wheat bran and dried carrot on the basic chemical composition of *Tenebrio molitor* larvae, the concentrations of selected lipid-soluble bioactive compounds, fatty acid composition, and selected biomarkers of oxidative status.

## 2. Materials and Methods

### 2.1. Experimental Material and Study Design

The experimental material consisted of mealworm (*Tenebrio molitor* L.) larvae at the penultimate larval instar. The larvae were housed in 30 plastic containers measuring 30 × 14.5 × 10 cm, fitted with ventilated lids and small containers containing cotton wool soaked with water to maintain adequate humidity. Each container was stocked with 200 g of larvae. The containers were randomly assigned to three dietary treatments, with ten replicates per treatment (*n* = 10). Each container of larvae constituted an experimental unit.

During the experiment, the larvae were maintained at a temperature of 22–24 °C, a relative humidity of 50–70%, and a 10 h light/14 h dark photoperiod. Before the experimental feeding period, the larvae were deprived of feed for 48 h. This 48 h feed deprivation period was used to reduce carry-over of the previous diet and to standardize gut contents before administration of the experimental diets. A 48 h fasting period has also been used in *T. molitor* to minimize the effects of the previous diet [32]. Subsequently, the experimental diets were provided once for a 48 h period at a rate of 150 g per container as a short-term feeding procedure preceding their intended use as feeder insects. Estimated feed consumption was calculated for each container as the difference between the dry matter of feed offered and that of the residual feed recovered after 48 h. Residual feed was separated from the larvae and frass by sieving and weighed, and its dry matter content was determined. Because complete correction for fine feed particles mixed with frass or other physical losses was not possible, this value should be regarded as an estimate of feed consumption rather than a direct measure of ingestion.

The experimental factor was the proportion of dried carrot in a wheat-bran-based diet. Wheat bran was selected as the control substrate because it is widely used as a basal feed in the rearing of *T. molitor*. Three diets were used: WB—100% wheat bran, DC50—50% dried carrot and 50% wheat bran, and DC75—75% dried carrot and 25% wheat bran. The chemical composition of the feed ingredients and experimental diets is presented in Table 1, while the contents of selected bioactive compounds are shown in Table 2, and the fatty acid composition of the experimental factors and diets is presented in Table 3.

The feed components were mixed in the appropriate proportions and subsequently pelleted to standardize the physical structure of the diets and minimize selective feed intake by the larvae. Pelleting was performed using a ZLSP-120B flat-die pellet mill (GEMCO, Anyang, China) equipped with a die with 4 mm openings. The pellets were produced in short batches, with the temperature of the device housing maintained below 35 °C to minimize thermal exposure during pelleting.

At the end of the 48 h feeding period, the larvae were frozen at −25 °C and transferred to the analytical laboratory approximately 24 h later. The larvae were not subjected to gut clearance immediately before analysis, as the aim of the study was to assess the nutritional value of whole larvae as feeder insects. Under practical reptile-feeding conditions, insects are consumed together with their gut contents; therefore, the determined chemical composition reflected the nutrients actually delivered to the target animal.

### 2.2. Chemical Analyses

Samples from each experimental unit were analyzed in duplicate, and the mean of the two technical determinations was used for statistical analysis. The nutrient contents of feed and mealworm larvae were determined using standard methods [33]. Dry matter content was determined by drying the samples in a laboratory dryer at 103 °C. Crude ash content was determined by sample mineralization in a muffle furnace (Czylok, Jastrzębie-Zdrój, Poland) at 600 °C. Total nitrogen content was determined by the Kjeldahl method using a FOSS TECATOR Kjeltec 2200 Auto Distillation Unit (FOSS Analytical AB, Höganäs, Sweden). Crude protein content in feed ingredients and experimental diets was calculated using a nitrogen-to-protein conversion factor of 6.25, whereas protein content in whole *Tenebrio molitor* larvae was estimated using a conversion factor of 4.76, as proposed by Janssen et al. [34]. Ether extract content was determined by the Soxhlet method using a FOSS SOXTEC SYSTEM 2043 (FOSS Analytical AB, Höganäs, Sweden). Crude fiber content was determined using a FOSS TECATOR Fibertec 2010 System (FOSS Analytical AB, Höganäs, Sweden). Organic matter content was calculated as the difference between dry matter and crude ash content, whereas nitrogen-free extract was calculated by subtracting crude protein, ether extract, and crude fiber from organic matter.

### 2.3. Content of Retinol, Carotenoids and Tocopherols

The concentrations of retinol and tocopherol homologues in feed were determined by High-Performance Liquid Chromatography in accordance with Polish Standards [35,36]. Analytical samples were saponified (50% KOH in ethanol *w*/*v*, 90 °C, 30′, reflux condenser) and extracted with ethanol and petroleum ether. The extracts (upper fraction—hydrophobic ether extract; lower fraction—hydrophilic) were rinsed with 10% (*w*/*v*) aqueous solution of NaCl, then with distilled water, dehydrated with anhydrous sodium sulfate and evaporated to dryness in the presence of nitrogen (N2). The residue was dissolved in 96% ethanol to determine the content of tocopherols and retinol and in n-hexane to determine the content of β-carotene and xanthophylls.

The concentrations of retinol and tocopherol homologues in yellow mealworm larvae were determined as described by Hőgberg et al. [37] and Xu [38]. Samples of whole larvae (1 g) were homogenized with 1.2 cm^3^ of 20% ascorbic acid in H_2_O (Ultra-Turrax T25 homogenizer, IKA Janke & Kunkel, Staufen, Germany) and saponified (375 g KOH, 750 cm^3^ H_2_O, 450 cm^3^ methanol, 80 °C, 15′). Ethanol (35% *v*/*v*) and NaCl (10% *w*/*v*) were added to cooled samples, which were extracted twice with n-hexane (2 × 4 cm^3^) and centrifuged (MPW-350R centrifuge, MPW Med. Instruments, Warsaw, Poland, 1500× *g*, 15′). 3 cm^3^ and 4 cm^3^ of the supernatant were collected successively. The combined extracts were evaporated to dryness under a stream of N_2_. The residue was dissolved in 1 cm^3^ of 96% ethanol to determine the content of tocopherols and retinol, and in n-hexane to determine the content of β-carotene and xanthophylls.

The levels of retinol and tocopherol homologues (α-, β-, γ-, and δ-tocopherol) in feed and yellow mealworm larvae were determined by reversed-phase high-performance liquid chromatography (RP-HPLC; SHIMADZU, Kyoto, Japan) on a Nucleosil C18 column, with methanol/water (95:5, *v*/*v*) as the mobile phase. Retinol was detected using a UV detector at 326 nm, whereas tocopherol homologues were detected using a fluorescence detector at an excitation wavelength of 295 nm and an emission wavelength of 330 nm. The flow rate was 1 cm^3^ min^−1^, and the injection loop volume was 20 µL. The concentrations of retinol and tocopherol homologues were estimated by comparing their peak areas with those of external standards: retinol (vitamin A alcohol, Sigma-Aldrich Chemie GmbH, Taufkirchen, Germany, No. R7632) and α-, β-, γ-, and δ-tocopherol standards from the Tocopherol Set (Merck KGaA, Darmstadt, Germany, No. 613424). The recoveries of α-tocopherol and retinol were 96% ± 2.8% and 89% ± 4.5%, respectively.

The hexane or petroleum ether extract of carotenoids, including β-carotene and xanthophylls, was filtered through a 0.22 µm PTFE syringe filter (30-SF-02, CHROMACOL Ltd., Welwyn Garden City, UK) and analyzed by RP-HPLC (SHIMADZU, Kyoto, Japan) on a Gemini C18 column (5 µm, 110 Å, 250 × 4 mm; Phenomenex, Torrance, CA, USA). The mobile phase consisted of methanol/tetrahydrofuran (95:5, *v*/*v*; HPLC grade, Sigma-Aldrich Chemie GmbH, Taufkirchen, Germany), the flow rate was 1 cm^3^ min^−1^, the detector wavelength was 450 nm, and the injection loop volume was 20 µL. The concentrations of β-carotene, lutein, and zeaxanthin were estimated by comparing their peak areas with those of external standards: β-carotene (β-carotene, provitamin A, Sigma-Aldrich Chemie GmbH, Taufkirchen, Germany, No. C4646; recovery: 85% ± 1.9%), lutein (xanthophyll–lutein, α-carotene-3,3′-diol, Sigma-Aldrich Chemie GmbH, Taufkirchen, Germany, No. X6250; xanthophyll recovery: 86% ± 6%), and zeaxanthin (β,β-carotene-3,3′-diol, Supelco, Sigma-Aldrich Chemie GmbH, Taufkirchen, Germany, No. 14681). The analysis was performed according to Rodriguez-Bernaldo de Quirós and Costa [39].

### 2.4. Fatty Acid Composition

Fatty acid composition was determined in samples of the experimental diets and yellow mealworm larvae after lipid extraction. The samples were ground and subjected to Soxhlet extraction according to AOAC [31]. The lipid extracts were then converted to fatty acid methyl esters (FAMEs) using the modified Peisker procedure [40]. For this purpose, an aliquot of the extracted fat was mixed with 1.5 cm^3^ of methanol/chloroform/concentrated sulfuric acid solution (100:100:1, *v*/*v*/*v*) and heated at 80 °C for 3 h. The resulting FAMEs were analyzed by gas chromatography using a VARIAN CP-3800 gas chromatograph (Varian Inc., Walnut Creek, CA, USA) equipped with a flame-ionization detector. Separation was performed on a capillary column measuring 50 m × 0.25 mm, with a film thickness of 0.25 µm. The following chromatographic conditions were applied: split injection mode, split ratio 50:1, injection volume 1 µL, injector temperature 225 °C, detector temperature 250 °C, column temperature 200 °C, helium carrier gas, and carrier gas flow rate of 1.2 cm^3^/min. Individual fatty acids were identified based on the retention times of fatty acid methyl ester standards (Sigma-Aldrich Chemie GmbH, Taufkirchen, Germany). The results were expressed as the percentage contribution of each fatty acid to the total fatty acid peak area. The analytical procedure was based on the method described by Peisker [39], with chromatographic conditions adapted from Kowalska et al. [41].

### 2.5. Antioxidant Enzyme Activity

The activities of antioxidant enzymes and lipid peroxidation status were determined in whole-body homogenates of yellow mealworm larvae. Briefly, whole larvae were homogenized under chilled conditions in phosphate buffer, and the homogenates were centrifuged. The resulting supernatants were used for biochemical analyses. Superoxide dismutase (SOD) and peroxidase (POX) activities were determined spectrophotometrically using commercial assay kits on an ACCENT-200 biochemistry analyzer (PZ Cormay S.A., Łomianki, Poland), according to the manufacturer’s instructions. Malondialdehyde (MDA) concentration was determined using a commercial Lipid Peroxidation (MDA) Assay Kit (Sigma-Aldrich, Merck KGaA, Darmstadt, Germany; catalog no. MAK568), according to the manufacturer’s instructions. The results were expressed as nmol MDA equivalents per g of fresh matter.

### 2.6. Statistical Analysis

Results are presented as mean values ± standard deviation (SD). The statistical analysis was based on 10 independent experimental units per treatment (*n* = 10). Normality of model residuals was assessed using the Shapiro–Wilk test, and homogeneity of variances was evaluated using Levene’s test. Differences among the dietary groups (WB, DC50, and DC75) were evaluated using one-way Welch’s ANOVA. For variables showing deviations from normality, the results were additionally verified using the Kruskal–Wallis test, which yielded the same conclusions regarding statistical significance. *p*-values from the global Welch’s ANOVA tests were adjusted using the Benjamini–Hochberg false discovery rate (FDR) procedure. For variables showing a significant global effect, pairwise comparisons were performed using Welch’s *t*-test with Holm-adjusted *p*-values. Differences were considered statistically significant at an adjusted *p* ≤ 0.05. All statistical analyses were performed using R software, version 4.5.1 [42].

## 3. Results

Estimated feed consumption was 88.4 ± 4.0, 90.5 ± 3.9, and 92.4 ± 3.7 g dry matter/container/48 h in the WB, DC50, and DC75 groups, respectively (Welch’s ANOVA, *p* = 0.101). All measurements reported for larvae refer to whole larvae, including gut contents.

The proximate composition of the larvae did not differ among the dietary groups (Table 4). In contrast, significant between-group differences were observed in the concentrations of bioactive compounds. As shown in Table 5, retinol concentration was higher in the DC75 group than in the other two groups. The concentrations of β-carotene, lutein, and zeaxanthin were highest in larvae from the DC75 group, intermediate in those from the DC50 group, and lowest in larvae fed the diet without dried carrot (DC75 > DC50 > WB). The highest concentrations of α-tocopherol and β-tocopherol were found in larvae from the WB group, whereas lower concentrations were observed in the DC50 and DC75 groups (WB > DC50 and DC75). The γ-tocopherol concentration was highest in the WB group, intermediate in the DC50 group, and lowest in the DC75 group, with all three groups differing significantly from one another. Overall, total tocopherol concentration was higher in larvae fed the WB diet alone than in those receiving diets containing dried carrot (WB > DC50 and DC75).

The proportion of myristic acid was higher in the WB group than in the DC75 group, whereas the proportion of the remaining saturated fatty acids (SFA) was higher in the DC50 and DC75 groups than in the WB group (Table 6). Among monounsaturated fatty acids, a lower proportion of palmitoleic acid was observed in the DC75 group (DC75 < WB and DC50). Similarly, the proportion of oleic acid was lower in the DC75 group than in the WB group. The proportion of the remaining monounsaturated fatty acids (MUFA) was higher in the DC75 group than in the WB group, with an intermediate value in the DC50 group. The most pronounced changes were observed for polyunsaturated fatty acids. The proportion of linoleic acid was higher in the DC75 group than in the WB group. Similarly, the proportion of α-linolenic acid increased with the increasing inclusion level of dried carrot in the diet, reaching the highest value in the DC75 group, an intermediate value in the DC50 group, and the lowest value in the WB group (DC75 > DC50 > WB). The total proportion of polyunsaturated fatty acids (PUFA) was higher in the DC75 group than in the WB group. In addition, the n-6/n-3 PUFA ratio decreased with the increasing inclusion level of dried carrot in the diet and was highest in the WB group, intermediate in the DC50 group, and lowest in the DC75 group (WB > DC50 > DC75).

The inclusion of dried carrot in the diet did not significantly affect antioxidant enzyme activity in mealworm larvae (Table 7). Peroxidase (POX) activity was numerically highest in the WB group, intermediate in the DC50 group, and lowest in the DC75 group; however, these differences were not statistically significant. Superoxide dismutase (SOD) activity also decreased numerically with the increasing inclusion level of dried carrot in the diet, but the differences among groups were not statistically significant. In contrast, significant between-group differences were observed in malondialdehyde (MDA) concentration. MDA concentration was higher in the WB group than in the DC75 group, whereas the DC50 group showed an intermediate value and did not differ significantly from either of the other groups.

## 4. Discussion

The inclusion of dried carrot markedly altered the composition of the experimental diets (Table 1); however, the 48 h feeding period did not result in significant changes in the proximate composition of whole larvae (Table 4). Similarly, Gorst et al. [18] reported that, following a 48 h gut-loading period, the concentrations of major nutrients in *Tenebrio molitor* larvae remained relatively stable for up to 12 h after removal of the gut-loading diet. Moreover, Liu et al. [27], whose larvae were fasted for 24 h before analysis, demonstrated that a longer, 4-week enrichment of mealworm diets with fresh plant materials, including carrot, also did not cause significant changes in the proximate composition of the larvae. The relative stability of most parameters of larval proximate composition may partly reflect the short duration of the experiment. Oonincx et al. [16] demonstrated that diet composition can affect insect development and chemical composition, although the magnitude of these effects varies among insect species and dietary treatments.

In contrast to proximate composition, the concentrations of lipophilic compounds were markedly modified by the inclusion of dried carrot in the diet (Table 5). The concentrations of β-carotene, lutein, and zeaxanthin increased with the increasing inclusion level of dried carrot, reaching the highest values in the DC75 group. These results show that increasing dried carrot inclusion at the expense of wheat bran increased carotenoid concentrations in whole larvae. Many carotenoids present in insects, which may subsequently be accumulated or partially modified metabolically, are derived from the diet [43]. This indicates that their presence in the insect body is largely dependent on diet composition. Baldacchino et al. [28], whose larvae were starved for 48 h before analysis, also reported substantial excretion of β-carotene and lycopene in mealworm frass, indicating that gut clearance may considerably reduce the amount of dietary carotenoids retained in larvae. Similarly, Liu et al. [27] noted that carrots are a source of carotenoids such as β-carotene, lutein, and zeaxanthin. It should be emphasized, however, that in the present experiment, the increase in carotenoid content reflects an increase in their content in whole larvae intended for use as feeder insects. The values obtained therefore include both compounds absorbed and deposited in the tissues and components present in the gut contents. This approach is practically justified because, in the feeding of reptiles and other insectivorous animals, larvae are consumed together with the contents of their gastrointestinal tract [11,13]. In this context, the results obtained provide a good reflection of the actual nutritional value of the larvae following the gut-loading procedure.

β-Carotene, as a compound with provitamin A activity, can be enzymatically cleaved to retinal and subsequently reduced to retinol or oxidized to retinoic acid [44]. However, carotenoid-to-retinoid metabolism in insect larvae differs from that described in vertebrates, and preformed vitamin A is generally present at very low or non-detectable levels before pupation [13]. In the present study, retinol was not detected in the feed components or experimental diets, whereas its concentration was higher in larvae from the DC75 group. This finding could be consistent with metabolic conversion of β-carotene; however, this interpretation should be treated with caution because whole larvae, including their gut contents, were analyzed, and the relative contributions of tissue deposition and residual intestinal contents could not be distinguished.

Total tocopherol content, as well as the contents of α- and β-tocopherol, was lower in larvae receiving diets containing dried carrot than in larvae fed wheat bran alone. Wheat bran is a source of vitamin E compounds, including tocopherols and tocotrienols [45]. Carrots also contain vitamin E compounds, although their levels are generally relatively lower [46]. Therefore, the lower tocopherol content observed in larvae fed the DC50 and DC75 diets is not inconsistent with the expected effect of carrot inclusion but most likely reflects the fact that, in the present experiment, dried carrot was a very good source of carotenoids but a poorer source of tocopherols than wheat bran.

The fatty acid profile obtained in the present study is consistent with previous observations for *Tenebrio molitor* larvae, in which oleic, linoleic, and palmitic acids are generally the predominant fatty acids. Riekkinen et al. [47] reported that C16:0, C18:1n9, and C18:2n6 are among the major fatty acids in mealworm larvae, whereas their relative proportions may depend on the dietary substrate used. However, the fatty acid profile of the larvae does not necessarily directly reflect the fatty acid profile of the diet, because insects may selectively absorb, accumulate, or metabolically modify individual fatty acids. The decrease in the proportion of oleic acid and the increase in the proportion of α-linolenic acid in the DC75 group may be related to the composition of the experimental diets, as dried carrot was characterized by a lower proportion of oleic acid and a higher proportion of α-linolenic acid than wheat bran. However, this effect was moderate, which may be attributable to the short feeding period. Of particular nutritional relevance was the reduction in the n-6/n-3 PUFA ratio in the groups receiving dried carrot. Oonincx et al. [16] reported high n-6/n-3 ratios in mealworm larvae and indicated that further dietary studies are needed to improve their fatty acid profile, as these larvae showed a greater tendency to retain n-6 than n-3 fatty acids. In the present study, dried carrot did not lead to a radical change in the lipid profile, but it significantly shifted the n-6/n-3 PUFA ratio in a favorable direction. This may indicate that even a short gut-loading period can improve selected indices of larval lipid quality.

Regarding oxidative status indicators, the inclusion of dried carrot did not significantly affect POX or SOD activity, although the activities of both enzymes were numerically lower in the DC50 and DC75 groups. The lack of significant differences in antioxidant enzyme activity may indicate that the 48 h feeding period did not constitute a stress stimulus sufficient to elicit a strong enzymatic response in the larvae. This is consistent with the findings of Liu et al. [27], who, after feeding mealworm larvae diets enriched with fresh plant materials, including carrot, for four weeks, did not observe a consistent enhancement of antioxidant activity in the larvae. In contrast, the concentration of MDA, an indicator of lipid peroxidation, was lower in the DC75 group than in the WB group. This may suggest that the higher intake of carotenoids and other bioactive compounds present in dried carrot contributed to a reduction in the intensity of oxidative processes, despite the absence of significant changes in SOD and POX activity. However, because dried carrot replaced wheat bran and the diets differed in overall composition, this effect cannot be attributed specifically to carotenoids or other bioactive compounds of carrot. Nevertheless, a potential contribution of carotenoids is biologically plausible because carotenoids can act as antioxidant and photoprotective compounds, and in animals they are generally accumulated from the diet or partially modified metabolically. As is well established, carotenoids can exert antioxidant effects, including the reduction of lipid peroxidation [43]. It should also be noted that tocopherol content was lower in the DC50 and DC75 groups than in the WB group, whereas α-tocopherol is a well-established lipophilic antioxidant that protects PUFA against lipid peroxidation. The lower MDA concentration despite the reduced tocopherol content may suggest that, under the present conditions, carotenoids and/or other components of dried carrot may have played a greater role than the tocopherol pool itself. However, this interpretation should be treated with caution because whole larvae, including gut contents, were analyzed and MDA concentration may be influenced by multiple factors.

The results obtained in the present study may contribute to a better understanding of the nutritional effects of short-term dried-carrot feeding in *T. molitor* larvae. The study has certain limitations, which may, however, pave the way for future research. First, dried carrot was introduced by replacing wheat bran, and the experimental diets were not nutritionally matched. Consequently, increasing dried carrot inclusion was accompanied by changes in protein, fat, fiber, fatty acid composition, and tocopherol content. Therefore, the observed responses should be interpreted as effects of increasing dried carrot inclusion at the expense of wheat bran rather than as effects attributable to individual bioactive compounds in carrot. Another limitation is that potential contaminants in the feed ingredients, including mycotoxins, pesticide residues, and toxic elements, were not quantified in the present study. Therefore, potential effects of unmeasured contaminants on the observed responses cannot be completely excluded. In addition, the initial 48 h feed deprivation may have influenced larval metabolic status; however, this procedure was applied equally to all experimental groups. Growth and developmental performance were also not assessed because the study focused on short-term nutritional modification of larvae prior to their intended use as feeder insects. Finally, because whole larvae were analyzed without gut clearance, the relative contributions of tissue-associated compounds and residual gut contents to the measured concentrations of carotenoids and retinol could not be distinguished. Future research extending the present experimental approach should therefore compare whole and gut-cleared larvae under the same short-term dried-carrot feeding conditions to better distinguish tissue-associated carotenoids and retinol from compounds present in residual gut contents.

## 5. Conclusions

After 48 h, the inclusion of dried carrot at 50% and 75% of the experimental diets did not affect the proximate composition of whole larvae, including their gut contents, but increased the concentrations of β-carotene, lutein, and zeaxanthin. The highest concentrations of these compounds were observed in larvae receiving the diet containing 75% dried carrot. In contrast, tocopherol concentrations decreased. The inclusion of dried carrot also modified the fatty acid profile of the larvae, decreasing the proportions of myristic, palmitoleic, and oleic acids, while increasing the proportions of linoleic and α-linolenic acids and total PUFA, and decreasing the n-6/n-3 PUFA ratio. No significant changes were observed in SOD or POX activity, whereas a lower MDA concentration was found in the DC75 group, which may indicate reduced lipid peroxidation.

## Figures and Tables

**Table 1:** Chemical composition of feed ingredients and experimental diets (%, as-is basis).

Component	Wheat Bran	Dried Carrot	Diets		
			WB	DC50	DC75
Dry matter	86.96	89.10	86.96	88.09	88.66
Crude ash	4.64	3.00	4.64	3.80	3.45
Organic matter	82.32	86.10	82.32	84.29	85.21
Crude protein	15.67	4.83	15.67	10.25	7.72
Crude fat	3.00	0.23	3.00	1.62	0.89
Crude fiber	10.82	3.16	10.82	7.04	5.12
Nitrogen-free extract	52.83	77.88	52.83	65.38	71.48

WB—100% wheat bran; DC50—50% dried carrot and 50% wheat bran; DC75—75% dried carrot and 25% wheat bran.

**Table 2:** Contents of retinol, selected carotenoids, and tocopherols in feed ingredients and experimental diets (mg/kg, as-is basis).

Compound	Wheat Bran	Dried Carrot	Diets		
			WB	DC50	DC75
Retinol	nd	nd	nd	nd	nd
β-carotene	0.60	329.95	0.60	161.04	241.63
Lutein	0.60	11.85	0.60	6.02	8.94
Zeaxanthin	0.05	1.92	0.05	0.97	1.42
α-tocopherol	5.41	4.23	5.41	4.49	4.31
β-tocopherol	0.30	0.09	0.30	0.17	0.13
ɣ-tocopherol	5.23	0.01	5.23	2.47	1.16
δ-tocopherol	0.55	<0.01	0.55	0.24	0.13
Total tocopherols	11.49	4.33	11.49	7.37	5.73

WB—100% wheat bran; DC50—50% dried carrot and 50% wheat bran; DC75—75% dried carrot and 25% wheat bran; nd—not detected, the limit of detection for retinol = 0.01 mg/kg.

**Table 3:** Fatty acid composition of feed ingredients and experimental diets (% of total fatty acids).

Fatty Acid	Wheat Bran	Dried Carrot	Diets		
			WB	DC50	DC75
C14:0 (myristic)	0.41	0.59	0.41	0.48	0.49
C16:0 (palmitic)	17.93	21.04	17.93	18.30	18.68
C18:0 (stearic)	2.22	2.93	2.22	2.31	2.38
Other SFA	1.38	2.42	1.38	1.48	1.61
Total SFA	21.94	26.98	21.94	22.57	23.16
C16:1 (palmitoleic)	0.28	0.47	0.28	0.33	0.36
C18:1 (oleic)	17.14	10.53	17.14	16.51	15.63
Other MUFA	1.71	2.05	1.71	1.71	1.73
Total MUFA	19.13	13.05	19.13	18.55	17.72
C18:2 (linoleic)	52.50	44.24	52.50	51.43	50.62
C18:3 (α-linolenic)	6.14	14.13	6.14	6.75	7.74
Other PUFA	0.29	1.60	0.29	0.70	0.76
Total PUFA	58.93	59.97	58.93	58.88	59.12
PUFA n-6/n-3	8.55	3.13	8.55	7.62	6.54

WB—100% wheat bran; DC50—50% dried carrot and 50% wheat bran; DC75—75% dried carrot and 25% wheat bran; SFA—saturated fatty acids; MUFA—monounsaturated fatty acids; PUFA—polyunsaturated fatty acids.

**Table 4:** Proximate composition of mealworm larvae (%, fresh matter basis) (mean ± SD).

Component	Dietary Treatments			*p*-Value *
	WB	DC50	DC75	
Dry matter	41.04 ± 1.58	41.61 ± 2.25	41.87 ± 1.75	0.562
Crude ash	1.60 ± 0.07	1.58 ± 0.13	1.58 ± 0.06	0.789
Organic matter	39.44 ± 1.59	40.03 ± 2.25	40.29 ± 1.76	0.562
Estimated protein content (N × 4.76)	17.11 ± 0.79	16.35 ± 1.71	16.17 ± 1.48	0.231
Crude fat	13.63 ± 0.28	13.88 ± 0.55	14.10 ± 0.48	0.073
Crude fiber	1.72 ± 0.07	1.63 ± 0.57	1.67 ± 0.09	0.435
Nitrogen-free extract	6.98 ± 1.33	8.17 ± 1.77	8.35 ± 1.48	0.131

*n* = 10 independent experimental units per treatment. WB—100% wheat bran; DC50—50% dried carrot and 50% wheat bran; DC75—75% dried carrot and 25% wheat bran. * FDR-adjusted *p*-value.

**Table 5:** Contents of retinol, selected carotenoids, and tocopherols in mealworm larvae (mg/kg fresh matter) (mean ± SD).

Compound	Dietary Treatments			*p*-Value *
	WB	DC50	DC75	
Retinol	0.08 ± 0.03 ^a^	0.09 ± 0.02 ^a^	0.13 ± 0.04 ^b^	0.032
β-carotene	1.36 ± 0.09 ^a^	4.14 ± 0.30 ^b^	5.08 ± 0.47 ^c^	<0.001
Lutein	0.10 ± 0.02 ^a^	0.40 ± 0.04 ^b^	0.60 ± 0.07 ^c^	<0.001
Zeaxanthin	0.03 ± 0.01 ^a^	0.15 ± 0.02 ^b^	0.25 ± 0.02 ^c^	<0.001
α-tocopherol	0.31 ± 0.09 ^a^	0.16 ± 0.01 ^b^	0.18 ± 0.04 ^b^	0.003
β-tocopherol	0.05 ± 0.01 ^a^	0.02 ± 0.01 ^b^	0.02 ± 0.01 ^b^	<0.001
ɣ-tocopherol	0.07 ± 0.01 ^a^	0.06 ± 0.01 ^b^	0.04 ± 0.01 ^c^	<0.001
δ-tocopherol	0.02 ± 0.01	0.01 ± 0.01	0.01 ± 0.01	0.113
Total tocopherols	0.45 ± 0.09 ^a^	0.25 ± 0.02 ^b^	0.25 ± 0.05 ^b^	<0.001

*n* = 10 independent experimental units per treatment. WB—100% wheat bran; DC50—50% dried carrot and 50% wheat bran; DC75—75% dried carrot and 25% wheat bran. * FDR-adjusted *p*-value. ^a–c^ Mean values within rows with no common superscripts are significantly different at *p* ≤ 0.05.

**Table 6:** Fatty acid composition of mealworm larvae (% of total fatty acids) (mean ± SD).

Fatty Acid	Dietary Treatments			*p*-Value *
	WB	DC50	DC75	
C14:0 (myristic)	0.83 ± 0.06 ^a^	0.79 ± 0.07 ^ab^	0.72 ± 0.06 ^b^	0.008
C16:0 (palmitic)	18.47 ± 0.69	18.18 ± 0.64	17.93 ± 0.61	0.266
C18:0 (stearic)	2.88 ± 0.17	2.83 ± 0.16	2.77 ± 0.17	0.435
Other SFA	1.79 ± 0.12 ^a^	2.01 ± 0.15 ^b^	2.06 ± 0.16 ^b^	0.003
Total SFA	23.97 ± 0.84	23.81 ± 0.80	23.48 ± 0.75	0.435
C16:1 (palmitoleic)	1.68 ± 0.12 ^a^	1.61 ± 0.11 ^a^	1.47 ± 0.10 ^b^	0.004
C18:1 (oleic)	40.43 ± 1.19 ^a^	39.56 ± 1.11 ^ab^	38.66 ± 1.04 ^b^	0.017
Other MUFA	3.82 ± 0.25 ^a^	4.08 ± 0.29 ^ab^	4.31 ± 0.32 ^b^	0.011
Total MUFA	45.93 ± 1.29	45.25 ± 1.21	44.44 ± 1.14	0.073
C18:2 (linoleic)	27.06 ± 0.91 ^a^	27.77 ± 0.95 ^ab^	28.64 ± 1.01 ^b^	0.015
C18:3 (α-linolenic)	1.81 ± 0.14 ^a^	2.10 ± 0.17 ^b^	2.39 ± 0.18 ^c^	<0.001
Other PUFA	1.23 ± 0.10 ^a^	1.07 ± 0.09 ^b^	1.05 ± 0.08 ^b^	0.003
Total PUFA	30.10 ± 1.05 ^a^	30.94 ± 1.09 ^ab^	32.08 ± 1.15 ^b^	0.008
PUFA n-6/n-3	14.95 ± 0.79 ^a^	13.22 ± 0.70 ^b^	11.98 ± 0.66 ^c^	<0.001

*n* = 10 independent experimental units per treatment. WB—100% wheat bran; DC50—50% dried carrot and 50% wheat bran; DC75—75% dried carrot and 25% wheat bran; SFA—saturated fatty acids; MUFA—monounsaturated fatty acids; PUFA—polyunsaturated fatty acids. * FDR-adjusted *p*-value. ^a–c^ Mean values within rows with no common superscripts are significantly different at *p* ≤ 0.05.

**Table 7:** Antioxidant enzyme activities (U/mg protein) and malondialdehyde concentration (nmol/g fresh matter) in mealworm larvae (mean ± SD).

Biomarker	Dietary Treatments			*p*-Value *
	WB	DC50	DC75	
POX	3.16 ± 0.46	2.97 ± 0.41	2.69 ± 0.37	0.092
SOD	54.60 ± 6.12	51.38 ± 5.84	48.72 ± 5.09	0.131
MDA	1.76 ± 0.22 ^a^	1.54 ± 0.20 ^ab^	1.41 ± 0.22 ^b^	0.017

*n* = 10 independent experimental units per treatment. WB—100% wheat bran; DC50—50% dried carrot and 50% wheat bran; DC75—75% dried carrot and 25% wheat bran. POX—peroxidase; SOD—superoxide dismutase; MDA—malondialdehyde. * FDR-adjusted *p*-value. ^a,b^ Mean values within rows with no common superscripts are significantly different at *p* ≤ 0.05.

## Data Availability

The data presented in this study are available upon request from the corresponding author.

## References

[1] Errico S., Spagnoletta A., Verardi A., Moliterni S., Dimatteo S., Sangiorgio P. (2022). *Tenebrio molitor* as a source of interesting natural compounds, their recovery processes, biological effects, and safety aspects. Compr. Rev. Food Sci. Food Saf..

[2] Syahrulawal L., Torske M.O., Sapkota R., Næss G., Khanal P. (2023). Improving the nutritional values of yellow mealworm *Tenebrio molitor* (Coleoptera: Tenebrionidae) larvae as an animal feed ingredient: A review. J. Anim. Sci. Biotechnol..

[3] Kavle R.R., Pritchard E.T.M., Bekhit A.E.-D.A., Carne A., Agyei D. (2022). Edible insects: A bibliometric analysis and current trends of published studies (1953–2021). Int. J. Trop. Insect Sci..

[4] Morales-Ramos J.A., Tomberlin J.K., Miranda C., Rojas M.G. (2024). Rearing methods of four insect species intended as feed, food, and food ingredients: A review. J. Econ. Entomol..

[5] Muñoz-Seijas N., Fernandes H., Domínguez J.M., Salgado J.M. (2025). Recent advances in biorefinery of *Tenebrio molitor* adopting green technologies. Food Bioprocess Technol..

[6] Yi L., van Boekel M.A.J.S., Boeren S., Lakemond C.M.M. (2016). Protein identification and in vitro digestion of fractions from *Tenebrio molitor*. Eur. Food Res. Technol..

[7] Zou X., Liu M., Li X., Pan F., Wu X., Fang X., Zhou F., Peng W., Tian W. (2024). Applications of insect nutrition resources in animal production. J. Agric. Food Res..

[8] Cooper J.E., Williams D.L. (2014). The feeding of live food to exotic pets: Issues of welfare and ethics. J. Exot. Pet Med..

[9] Valdés F., Villanueva V., Durán E., Campos F., Avendaño C., Sánchez M., Domingoz-Araujo C., Valenzuela C. (2022). Insects as feed for companion and exotic pets: A current trend. Animals.

[10] Finke M.D. (2002). Complete nutrient composition of commercially raised invertebrates used as food for insectivores. Zoo Biol..

[11] Finke M.D. (2003). Gut loading to enhance the nutrient content of insects as food for reptiles: A mathematical approach. Zoo Biol..

[12] Latney L., Clayton L.A. (2014). Updates on amphibian nutrition and nutritive value of common feeder insects. Vet. Clin. North Am. Exot. Anim. Pract..

[13] Oonincx D.G.A.B., Finke M.D. (2023). Insects as a complete nutritional source. J. Insects Food Feed.

[14] Livingston S., Lavin S.R., Sullivan K., Attard L., Valdes E.V. (2014). Challenges with effective nutrient supplementation for amphibians: A review of cricket studies. Zoo Biol..

[15] van Broekhoven S., Oonincx D.G.A.B., van Huis A., van Loon J.J.A. (2015). Growth performance and feed conversion efficiency of three edible mealworm species (Coleoptera: Tenebrionidae) on diets composed of organic by-products. J. Insect Physiol..

[16] Oonincx D.G.A.B., van Broekhoven S., van Huis A., van Loon J.J.A. (2015). Feed conversion, survival and development, and composition of four insect species on diets composed of food by-products. PLoS ONE.

[17] Grau T., Vilcinskas A., Joop G. (2017). Sustainable farming of the mealworm *Tenebrio molitor* for the production of food and feed. Z. Naturforsch. C.

[18] Gorst V.M., Mitchell K., Whitehouse-Tedd K.M. (2015). Effect of post-gut loading time on the macro-nutrient content of three feeder invertebrate species. J. Zoo Aquar. Res..

[19] Boykin K., Bitter A., Mitchell M.A. (2021). Using a commercial gut loading diet to create a positive calcium to phosphorus ratio in mealworms (*Tenebrio molitor*). J. Herpetol. Med. Surg..

[20] Meléndez-Martínez A.J. (2019). An overview of carotenoids, apocarotenoids, and vitamin A in agro-food, nutrition, health, and disease. Mol. Nutr. Food Res..

[21] Milani A., Basirnejad M., Shahbazi S., Bolhassani A. (2017). Carotenoids: Biochemistry, pharmacology and treatment. Br. J. Pharmacol..

[22] Kamel D.G., Hammam A.R.A., Nagm El-Diin M.A.H., Awasti N., Abdel-Rahman A.M. (2023). Nutritional, antioxidant, and antimicrobial assessment of carrot powder and its application as a functional ingredient in probiotic soft cheese. J. Dairy Sci..

[23] Ahmad T., Cawood M., Iqbal Q., Ariño A., Batool A., Tariq R.M.S., Azam M., Akhtar S. (2019). Phytochemicals in *Daucus carota* and their health benefits—Review article. Foods.

[24] Sharma K.D., Karki S., Thakur N.S., Attri S. (2012). Chemical composition, functional properties and processing of carrot—A review. J. Food Sci. Technol..

[25] Narwojsz A., Sawicki T., Piłat B., Tańska M. (2025). Effect of heat treatment methods on color, bioactive compound content, and antioxidant capacity of carrot root. Appl. Sci..

[26] Mattioli S., Paci G., Fratini F., Dal Bosco A., Tuccinardi T., Mancini S. (2021). Former foodstuff in mealworm farming: Effects on fatty acids profile, lipid metabolism and antioxidant molecules. LWT.

[27] Liu C., Masri J., Perez V., Maya C., Zhao J. (2020). Growth performance and nutrient composition of mealworms (*Tenebrio molitor*) fed on fresh plant materials-supplemented diets. Foods.

[28] Baldacchino F., Spagnoletta A., Lamaj F., Vitale M.L., Verrastro V. (2023). First Optimization of Tomato Pomace in Diets for *Tenebrio molitor* (L.) (Coleoptera: Tenebrionidae). Insects.

[29] Rovai D., Ortgies M., Amin S., Kuwahara S., Schwartz G., Lesniauskas R., Garza J., Lammert A. (2021). Utilization of Carrot Pomace to Grow Mealworm Larvae (*Tenebrio molitor*). Sustainability.

[30] Dreassi E., Cito A., Zanfini A., Materozzi L., Botta M., Francardi V. (2017). Dietary fatty acids influence the growth and fatty acid composition of the yellow mealworm *Tenebrio molitor* (Coleoptera: Tenebrionidae). Lipids.

[31] Mancini S., Fratini F., Turchi B., Mattioli S., Dal Bosco A., Tuccinardi T., Nozic S., Paci G. (2019). Former foodstuff products in *Tenebrio molitor* rearing: Effects on growth, chemical composition, microbiological load, and antioxidant status. Animals.

[32] Mamtimin T., Han H., Khan A., Feng P., Zhang Q., Ma X., Fang Y., Liu P., Kulshrestha S., Shigaki T. (2023). Gut microbiome of mealworms (*Tenebrio molitor* larvae) show similar responses to polystyrene and corn straw diets. Microbiome.

[33] Horwitz W., Latimer G.W. (2006). Official Methods of Analysis of AOAC International.

[34] Janssen R.H., Vincken J.-P., van den Broek L.A.M., Fogliano V., Lakemond C.M.M. (2017). Nitrogen-to-protein conversion factors for three edible insects: *Tenebrio molitor*, *Alphitobius diaperinus*, and *Hermetia illucens*. J. Agric. Food Chem..

[35] (2002). Animal Feeding Stuffs—Determination of Vitamin A Content—Method Using High-Performance Liquid Chromatography.

[36] (2002). Animal Feeding Stuffs—Determination of Vitamin E Content—Method Using High-Performance Liquid Chromatography.

[37] Högberg A., Pickova J., Babol J., Andersson K., Dutta P.C. (2002). Muscle lipids, vitamins E and A, and lipid oxidation as affected by diet and RN genotype in female and castrated male Hampshire crossbreed pigs. Meat Sci..

[38] Xu Z. (2008). Comparison of extraction methods for quantifying vitamin E from animal tissues. Bioresour. Technol..

[39] Rodriguez-Bernaldo de Quirós A., Costa H.S. (2006). Analysis of carotenoids in vegetable and plasma samples: A review. J. Food Compos. Anal..

[40] Peisker K.V. (1964). A rapid semi-micro method for preparation of methyl esters from triglycerides using chloroform, methanol, sulphuric acid. J. Am. Oil Chem. Soc..

[41] Kowalska D., Strychalski J., Zwoliński C., Gugołek A., Matusevicius P. (2020). The effect of mixture of rapeseed meal, white lupin seed, and pea seed in rabbit diets on performance indicators and fatty acid profile of meat and fat. Kafkas Univ. Vet. Fak. Derg..

[42] R Core Team (2025). R: A Language and Environment for Statistical Computing.

[43] Maoka T. (2020). Carotenoids as natural functional pigments. J. Nat. Med..

[44] Harrison E.H. (2022). Carotenoids, β-apocarotenoids, and retinoids: The long and the short of it. Nutrients.

[45] Górnaś P., Radenkovs V., Pugajeva I., Soliven A., Needs P.W., Kroon P.A. (2016). Varied composition of tocochromanols in different types of bran: Rye, wheat, oat, spelt, buckwheat, corn, and rice. Int. J. Food Prop..

[46] Luby C.H., Vernon R., Maeda H.A., Goldman I.L. (2015). Lack of correspondence between experimentally determined values of vitamin E in carrot (*Daucus carota* L.) and those reported in the USDA National Nutrient Database. HortScience.

[47] Riekkinen K., Väkeväinen K., Korhonen J. (2022). The effect of substrate on the nutrient content and fatty acid composition of edible insects. Insects.

